# Does subcutaneous administration of recombinant human erythropoietin increase thrombotic events in total hip arthroplasty? A prospective thrombelastography analysis

**DOI:** 10.1186/s13018-020-02083-w

**Published:** 2020-11-19

**Authors:** Ru-xin Ruan, Chao-wen Bai, Le Zhang, Chao-ran Huang, Sheng Pan, Xing-chen Zhang, Zheng-ya Zhu, Xin Zheng, Kai-jin Guo

**Affiliations:** Department of Orthopaedics, The Affiliated Hospital of Xuzhou Medical University, No. 99, Huaihai West Road, Xuzhou, Jiangsu Province 221000 China

**Keywords:** Recombinant human erythropoietin, Total hip arthroplasty, Thrombotic events, Thrombelastography, Perioperative anemia

## Abstract

**Background:**

Anemia is one of severe complications in the perioperative period of total hip arthroplasty (THA). Erythropoietin (EPO) has been considered to improve patients’ anemia state, but its efficiency and safety remains controversial.

**Methods:**

A total of 152 patients who underwent total hip arthroplasty from January 2017 to March 2019 were randomized to 2 groups. Recombinant human erythropoietin (rHu-EPO) group was treated with rHu-EPO subcutaneous injection 10000 IU after operation and once daily in the next week, while control group was treated with none extra treatment. Routine hematologic examination and thrombelastography (TEG) performed at different time point respectively. Doppler ultrasound for bilateral lower limbs was performed 1 day before surgery and 7 days after surgery. Auxiliary examination outcomes, blood transfusions outcomes, and postoperative complications were recorded as assessment indicators.

**Results:**

The difference in the relevant indexes of traditional coagulation and TEG values between two groups were not significantly. No significant difference was observed in the incidence of thromboembolism events and other complications between two groups during postoperative period. The amount of intraoperative blood loss was similar between the two groups. However, the postoperative use and dosage of allogeneic blood in the rHu-EPO group were lower than those in the control group. The hemoglobin and hematocrit level in the rHu-EPO group were higher than that in the control group after surgery.

**Conclusion:**

Postoperative subcutaneous injection of rHu-EPO can improve hematological anemia-related parameters, reduce the use and dosage of allogeneic blood transfusions (ABTs), and has no significant influence on the formation of thrombosis and other complications in patients undergoing total hip arthroplasty in short term.

## Introduction

Total hip arthroplasty (THA) is considered to be distinctly advantageous for various end-staged hip diseases [1, 2]. Perioperative anemia is a common problem in major orthopedic surgery, concerning approximately 15 to 25% of patients in the preoperative period and as much as 80% in the postoperative period [3]. Patients undergoing THA are always plagued with anemia due to blood loss [4] and perioperative anemia may lead to an increased morbidity and mortality [5]. Additional allogenic blood transfusions (ABTs) are an effective method to improve anemia state but could increase risks of virus transmission, transfusion reactions, and possible immunosuppression [6, 7]. Therefore, plenty of strategies are used in the perioperative period of THA to reduce ABT requirements and the application of hematopoietic agents has proven effective [8–10].

Erythropoietin (EPO) is one of the erythropoiesis-stimulating drugs, which promotes hematopoietic stem cells differentiate into red blood cell to improve the anemia state of patients. Its artificial synthetic recombinant human erythropoietin (rHu-EPO) is used clinically as exogenous human erythropoietin and has similar function with EPO. The benefits of perioperative use of rHu-EPO in major orthopedic procedures have been well established [11, 12]. The major misgiving is the application of rHu-EPO could cause venous thromboembolism (VTE). In a small-scale, double-blind, placebo-controlled study, EPO increases the incidence of thrombosis by 15% among healthy male volunteers [13]. However, studies associated with the effect of rHu-EPO on coagulation function of patients undergoing THA are still lacking.

Nowadays, traditional plasma-based coagulation test is routinely used to monitor the perioperative clotting status of patients with THA [14]. Compared with traditional coagulation test, thrombelastography (TEG) evaluates the whole process of blood coagulation as a continuous graph from the beginning of clot formation to fibrinolysis and has a higher sensitivity on detecting abnormal blood state. This technique has been gradually used in orthopedic surgery to direct individualized anticoagulant therapy [15, 16].

In this study, we used TEG to evaluate the safety of rHu-EPO in combination with routine coagulation tests and color Doppler ultrasound at different times during perioperative period. The other purpose of this study is to reconfirm the efficacy of rHu-EPO subcutaneous administration on improving patients’ anemia state.

## Materials and methods

### Patient selection and study design

After the hospital ethics committee approval, a prospective and randomized-controlled study was conducted in patients undergoing cementless unilateral primary THA from January 2017 to March 2019. Every participator enrolled was given a written, informed consent. Patients who were 18 years or older and diagnosed as osteoarthritis or osteonecrosis of femoral head were recruited in this study. Patients were excluded if they (1) were contraindicated to rHu-EPO; (2) had lower extremity deep venous thrombosis (DVT), pulmonary embolism or myocardial infarction and receive anticoagulant therapy or antiplatelet treatment recently; (3) had severe liver diseases, uncontrolled hypertension, clinically significant impairment of renal function, or any other organ insufficiencies; (4) preoperative Hb< 90 g/l; (5) had a history of hip fracture or required additional osteotomy during THA. Finally, 152 patients were meeting the inclusion criteria and randomized into 2 groups. The patients' characteristics details were shown in Table 1.

**Table 1:** Baseline characteristics of patients

	rHu-EPO group (*n* = 74)	Control group (*n* = 78)	*P* value
Age (years)	66.2 ± 10.9	65.7 ± 9.1	0.773
Female sex	44(59.5%)	40(51.3%)	0.311
Weight (kg)	65.9 ± 7.3	65.5 ± 9.1	0.805
Body mass index (kg/m^2^)	23.8 ± 3.7	23.7 ± 3.6	0.958
Harris score	44.84 ± 11.87	43.53 ± 10.90	0.479
Comorbidity			
Hypertension Diabetes mellitus	39(52.7%) 3(4.1%)	45(57.7%) 9(11.5%)	0.625 0.132

^*^P < 0.05

### Surgical approach and perioperative managements

All THAs were performed through a posterolateral incision by the same surgeon under general anesthesia. Patients in both groups received a 15 mg/kg loading dose intravenous infusion tranexamic acid 30 min before incision and the same dose 3 h after closing the incision. A drainage tube was used in the first 24 h after operation and retrieved on the morning of day 2 postoperatively. Color Doppler ultrasound of both lower limbs was performed in all patients 1 day before the surgery and postoperative day 7 (POD 7) to screen for DVT. Intravenous iron supplementation was administered in combination with rHu-EPO. Patients in the rHu-EPO group received 10000 IU of rHu-EPO injections (Epogen, Sansheng, Shenyang, China) subcutaneously and 200 mg iron sucrose (Ferrous Saccharose, Hengsheng, Nanjing, China) diluted with 250 ml normal saline intravenously 3 hours after the operation. The same dose of subcutaneous injections rHu-EPO was given once daily and intravenous iron sucrose was given on every other day within a week (POD 2, POD 4, and POD 6). Hb, HCT, and platelet count were examined on preoperative day 1, POD 1, POD 4, and POD 7. Traditional coagulation test and TEG (TEG model 5000, Haemoscope Corporation, USA) were examined on preoperative day 1, POD 1 and POD 7. Ten milligrams of rivaroxaban were prescribed (Xarelto, Bayer, Leverkusen, Germany) orally daily as postoperation anticoagulant prophylaxis and the anticoagulant treatment lasted for a minimum of 35 days. The same rehabilitation treatments containing intravenous prophylactic antibiotics, wound care, and early functional exercises were provided equally to all patients during the stay in hospital.

### Transfusion protocol

A common restrictive transfusion protocol was established. All patients with any Hb≤ 70 g/L received RBC transfusions independently of symptoms. Patients with hemoglobin (Hb) level ranging from 71 to 100 g/L received red blood cells (RBC) transfusions depended on the clinical symptoms of anemia or uncontrolled hypovolemia (hypotension, tachycardia, tachypnea, dizziness, etc.). 1 U RBC was given for each transfusion until the Hb> 90 g/L or the acute anemia symptoms disappeared. We reexaminated these patients and decided to give another transfusion on the basis of their Hb level and clinical symptoms. No patient donated or received autologous blood.

### Outcome measures

The primary outcomes were the hematological parameters of routine coagulation test and TEG. Routine blood examination and transfusion outcomes like the number of patients transfused and the number of transfusions per patient were recorded as secondary outcomes. Thromboembolism events and other complications(periprosthetic joint or superficial wound infections, large subcutaneous ecchymosis, fatigue and dizziness, etc.)were also noted.

### Statistical analysis

The Kolmogorov-Smirnov test was used to determine whether measured and calculated parameters were normally distributed. Continuous variables were expressed as means ± standard deviations. Categorical variables were described using counts and percentages. Continuous variables between rHu-EPO and control groups were compared using independent-samples*t* test. Overall differences in the continuous variables at different time points were assessed by one-way analysis of variance. Pearson’s chi-squared test or Fisher’s exact test was used to compare the proportion of categorical variables, as appropriate. All of the data were statistically analyzed using SPSS 20.0 software (IBM Corp, Armonk, New York, USA). *P* < 0.05 was considered as statistically significant.

## Results

### Patient demographics

**Fig. 1:**
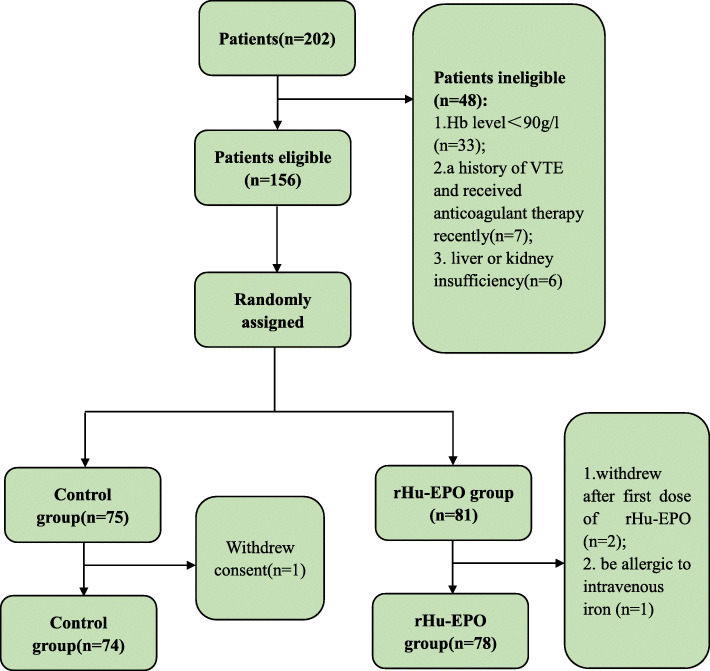
The flow chart of patient enrollment

A total of 202 patients were evaluated for eligibility. Of these, 33 patients’ Hb were inferior to the minimum value of the admittance. Seven patients had a history of VTE and received anticoagulant therapy recently and 6 patients has liver or kidney insufficiency. Finally, 156 patients met the inclusion criteria and were randomized into the rHu-EPO group and the control group according to a computer-generated random sequence. In addition, 2 patients appearing hypertension and headaches after first dose of rHu-EPO and 1 patient suffering from gastrointestinal symptoms after receiving intravenous iron were excluded. Only one patient in control group withdrew the consent for private excuse during the whole trail (Fig. 1).

### Parameters of blood coagulation test

D-dimer and FDP increased significantly after the procedure. The mean APTT and PT had a highest mean value on POD 1. The values would then shorten but remain higher than that in preoperative period. Nevertheless, no significant differences in D-dimer, FDP, APTT, PT, TT, FIB, and INR were observed between the two groups at different time points (Table 2).

**Table 2:** Traditional coagulation assessment at different time points

Indexes	Time points	rHu-EPO group (*n* = 74)	Control group (*n* = 78)	*P* value
FDP (mg/l)	Pre-op POD1 POD7	3.38 ± 1.16 13.34 ± 9.85 15.09 ± 3.97	3.47 ± 2.01 15.95 ± 14.00 16.16 ± 4.25	0.727 0.189 0.110
D-dimer(ng/ml)	Pre-op POD1 POD7	1.18 ± 0.54 4.08 ± 1.75 4.77 ± 1.70	1.22 ± 0.74 4.47 ± 2.33 5.33 ± 2.42	0.710 0.241 0.105
PT(s)	Pre-op POD1 POD7	11.64 ± 0.74 12.83 ± 1.14 12.24 ± 1.16	11.85 ± 0.95 13.05 ± 0.85 12.54 ± 1.06	0.141 0.163 0.099
APTT(s)	Pre-op POD1 POD7	28.61 ± 3.34 31.80 ± 5.71 28.97 ± 3.41	28.81 ± 3.63 33.30 ± 5.48 30.02 ± 3.50	0.720 0.098 0.063
TT(s)	Pre-op POD1 POD7	16.98 ± 0.93 15.86 ± 1.05 15.55 ± 0.77	17.24 ± 1.29 16.20 ± 1.22 15.79 ± 0.97	0.161 0.069 0.094
FIB(g/l)	Pre-op POD1 POD7	3.52 ± 1.09 3.64 ± 1.01 4.82 ± 1.00	3.31 ± 0.95 3.84 ± 0.84 5.01 ± 1.03	0.204 0.176 0.268
INR	Pre-op POD1 POD7	1.01 ± 0.08 1.13 ± 0.17 1.06 ± 0.10	1.03 ± 0.084 1.15 ± 0.11 1.09 ± 0.09	0.062 0.261 0.066

*P < 0.05, *Pre-op* pre-operation, *POD1* postoperative day 1, *POD7* postoperative day 7, *FDP* fibrin degradation products, *PT* prothrombin time, *APTT* activated partial thromboplastin time, *TT* thrombin time, *FIB* fibrinogen, *INR* international normalized ratio

### TEG assessment

Change of TEG parameters during perioperative period was shown in Table 3. In general, hypercoagulability appears during perioperative period. *K* value kept decreasing until POD7. Postoperative Alpha value was higher than the preoperative period and reached the maximum value on POD7. MA value is lowest on POD 1 and then increasing, even higher than preoperative period. However, there were no statistically significant differences in TEG variables we recorded between the two groups on POD1 and POD7.

**Table 3:** Assessment of TEG at different time points

Indexes	Time points	rHu-EPO group (*n* = 74)	Control group (*n* = 78)	*P* value
R(min)	Pre-op POD1 POD7	5.95 ± 1.71 5.73 ± 2.04 5.49 ± 1.39	5.98 ± 1.37 5.62 ± 1.48 5.66 ± 1.14	0.886 0.695 0.403
K(min)	Pre-op POD1 POD7	2.12 ± 0.77 2.11 ± 1.08 1.77 ± 0.47	2.39 ± 1.05 2.31 ± 1.27 1.60 ± 0.65	0.072 0.283 0.069
Alpha(°)	Pre-op POD1 POD7	59.61 ± 6.36 63.03 ± 10.65 66.01 ± 7.31	59.05 ± 11.05 61.77 ± 10.73 66.80 ± 8.67	0.704 0.468 0.545
MA(mm)	Pre-op POD1 POD7	60.08 ± 5.77 57.31 ± 7.46 64.10 ± 2.13	59.01 ± 5.67 58.37 ± 6.17 65.11 ± 7.88	0.254 0.341 0.286
CI	Pre-op POD1 POD7	− 0.43 ± 1.54 − 0.48 ± 3.42 0.73 ± 0.63	− 0.66 ± 2.86 − 0.32 ± 2.70 0.90 ± 2.00	0.540 0.746 0.492

*P < 0.05, *Pre-op* pre-operation, *POD1* postoperative day 1, *POD7* postoperative day 7, *R* reaction time, *MA* maximum amplitude, *CI* coagulation index

### The risk of VTE and other complications

Three patients in the control group and 5 patients in the rHu-EPO group were detected to have DVT. The proportion of thromboembolic events in the rHu-EPO group (6.4%) was not significantly different from that in the control group (4.1%) (*P* > 0.05). One patient in control group had a high fever after received ABT and subsided after the administration of sensitive antibiotics. Other notable complications were not found among these patients.

### Change of hemoglobin level and platelet count

**Fig. 2:**
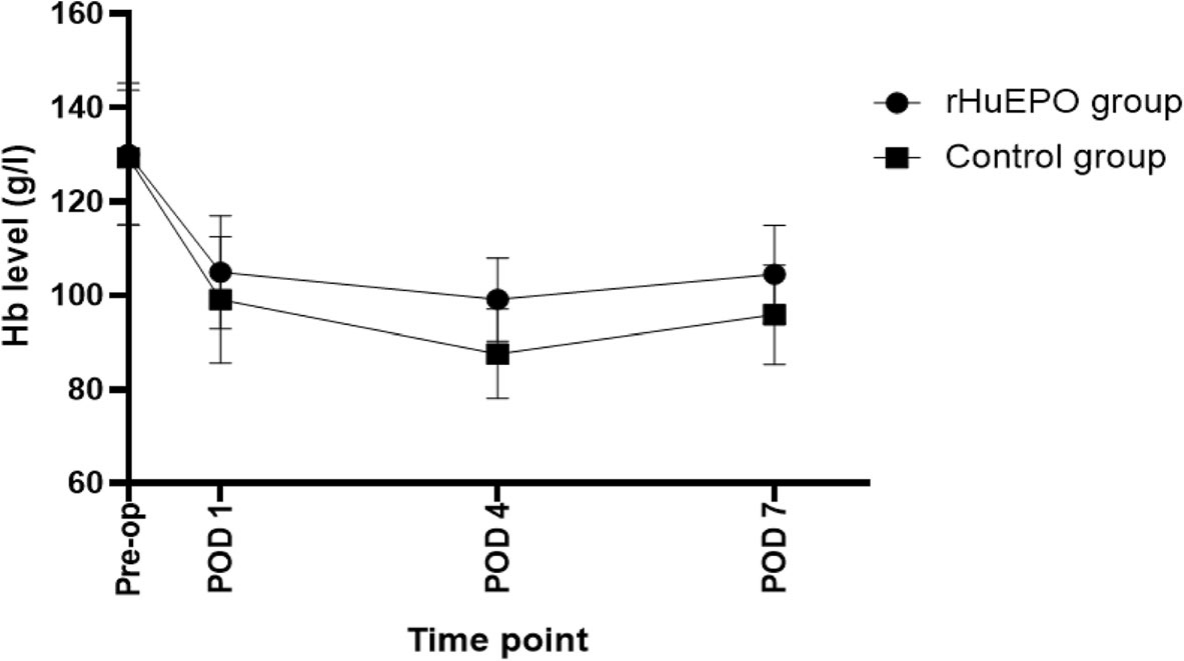
Hb level at different time point

**Fig. 3:**
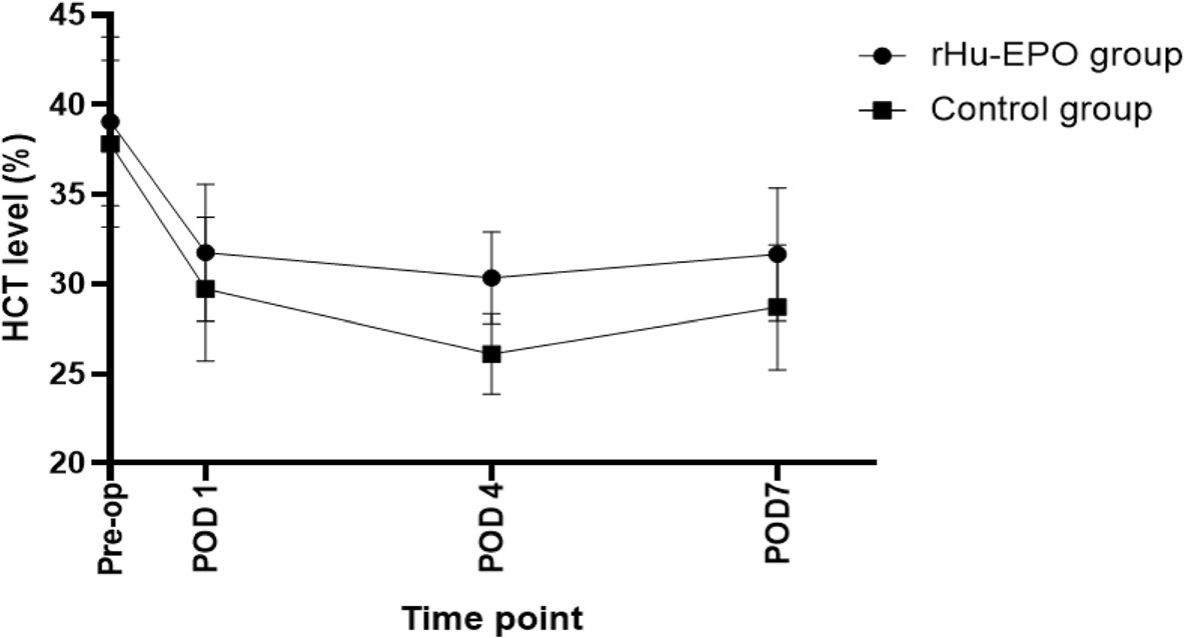
HCT level at different time point

**Fig. 4:**
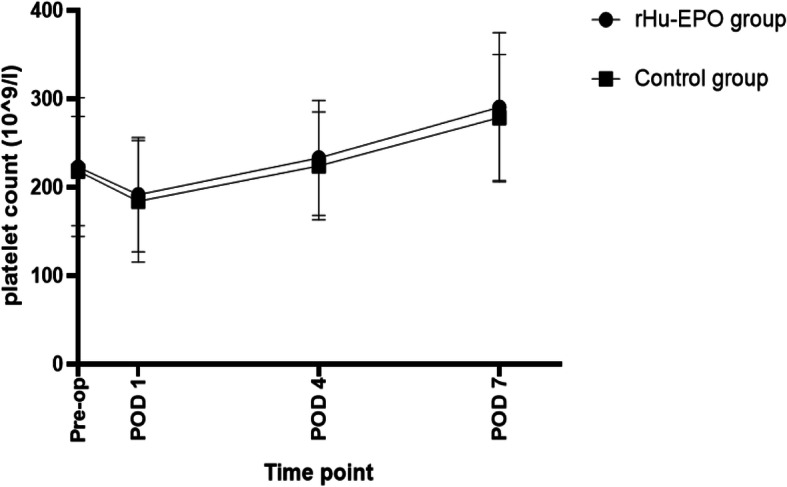
Platelet count at different time point

In Table 4, indexes associated with blood cells in the two groups decreased significantly on the first day after surgery. Hb and HCT on POD 4 and POD 7 were lower than that on POD 1 among all patients and were lowest on POD 4 (*P* < 0.05) as shown in Figs. 2 and 3. The change indicated patients had a recessive blood loss during postoperative period. Compared with the control group, patients in the rHu-EPO group had higher Hb and HCT level at each postoperative time point. Platelet count was lowest on POD 4 and then increased, even higher than preoperative state (Fig. 4).

**Table 4:** Routine blood examination indexes at different time points

Indexes	Time points	rHu-EPO group(*n* = 74)	Control group(*n* = 78)	*P* value
Hb(g/l)	Pre-op POD1 POD4 POD7	130.03 ± 15.15 104.90 ± 12.09 99.14 ± 8.81 104.47 ± 10.38	129.31 ± 14.29 99.05 ± 13.42 87.56 ± 9.44 95.88 ± 10.56	0.764 0.006* <0.001* <0.001*
HCT (%)	Pre-op POD1 POD4 POD7	39.07 ± 4.70 31.74 ± 3.82 30.34 ± 2.56 31.65 ± 3.71	37.83 ± 4.65 29.72 ± 4.01 26.10 ± 2.25 28.70 ± 3.48	0.103 0.002* <0.001* <0.001*
PLT (10^9^/l)	Pre-op POD1 POD4 POD7	222.68 ± 78.43 191.60 ± 64.59 233.08 ± 65.06 290.42 ± 84.46	218.37 ± 61.95 184.00 ± 68.96 224.01 ± 61.27 278.82 ± 71.58	0.707 0.485 0.378 0.362

^*^P < 0.05, *Pre-op* pre-operation, *POD1* postoperative day 1, *POD7* postoperative day 7, *RBC* red blood cell, *Hb* hemoglobin, *HCT* hematocrit, *PLT* platelet

### Requirements of allogenic blood

The amount of intraoperative blood loss was similar between the two groups. The rate of ABT was 23.1% (18 of 78 patients) in the control group and 9.5% (7 of 74 patients) in the rHu-EPO group (*P* = 0.029*). The mean volume of allogeneic blood required among patients who needed transfusion was 1.86 ± 0.69 U in rHu-EPO group and 3.50 ± 1.95 U in control group (*P* = 0.042*).

## Discussion

THA is associated with considerable blood loss and the subsequent need for ABTs [17]. Perioperative bleeding ranges from 900 to 1200 mL in primary THAs and leads to an apparent drop in the Hb level [18]. In our study, we found a higher level of anemia-related indexes and less exposure to ABT in the rHu-EPO group compared with patients in control group. Consistent with previous studies [19–21], this result further demonstrated rHu-EPO can minimize the need for ABT and decrease the average required volume of ABT after THA. We also recorded the change of routine coagulation and TEG parameters and found that hypercoagulability develops during the perioperative period. However, no significant difference of routine coagulation and TEG parameters were observed between rHu-EPO group and control group and the incidence of VTE between two groups was similar.

EPO is a hypoxia-induced hormone produced by the kidney and fetal liver which can stimulate hematopoiesis in the bone marrow. Since obtained Food and Drug Administration (FDA) license for clinical use, EPO and its analogs rHu-EPO have been used for treatment of the anemias caused by chronic renal failure, malignancies and proved to be effective [22, 23]. In recent years, EPO has captured the attention of surgeons for its excellent efficacy in improving perioperative anemia caused by massive blood loss [24].

The main concern in regard to rHu-EPO and other hematopoietic agents is the potentially increased risk of thrombotic events because the increased RBC could affect patients’ blood viscosity and hemodynamics [25–28]. No association was found between EPO administration and thromboembolic events according to a randomized, controlled trail [29]. It is hypothesized that the perioperative application of EPO does not increase the risk of VTE. The increased number of RBC may not reach the extent to VTE as patients were in an anemia status themselves after THA. However, the clotting function of patients undergoing THA with the administration of rHu-EPO still needs to be explored. Therefore, we combined traditional coagulation test with TEG to monitor perioperative blood hypercoagulability.

The significant increase of D-dimer and FDP suggested that postoperative patients had a higher blood coagulation than before. The *R* value represents the activity of coagulation factors in intrinsic and extrinsic pathways and has high significant relevance to APTT and PT. Van et al. [30] considered patients with postoperative DVT have shorter *R* value than patients without DVT because of the activation of coagulation factors at the beginning after operation. We did find postoperative *R*-time was shorter than preoperative *R*-time but no significant difference was found between the two groups, indicating that exogenous rHu-EPO injection did not affect coagulation factors. In the present study, *K* value and alpha angle reflect the clotting rate and linearly correlate with FIB [31]. FIB is an essential blood clotting protein, which in high does is an indicator of future VTE. These three values were changing toward a high coagulation trend but no statistically significant difference was found between the two groups on POD 1 and POD7, indicating that exogenous rHu-EPO injection had fewer influence on fibrinogen.

MA value is mainly related to the number and functional status of platelets and its change was basically consistent with platelet count according to previous experimental result [31]. Both of MA and PLT decreased in the first few days after operation, and then rebound, even higher than the preoperative level on POD7. It is related to the activation of platelet at later stage after operation, but the difference between two groups was not significant. Cotton et al. [32] believed that MA value could predict pulmonary embolism. When the MA value > 65 mm, the probability of pulmonary embolism increased by 3.5 times. MA values of the control group and the rHu-EPO group were (65.11 ± 7.88) mm and (64.10 ± 2.13) mm on POD 7 respectively. Therefore, patients remained in a hypercoagulable state after THA. We used oral rivaroxaban as a preventative anticoagulant therapy and the treatment would prolong to 3 months for patients with DVT. Under this anticoagulant condition, there was no significant difference of thrombotic events between two groups.

There is still no consensus regarding the administration of EPO currently. Zhao et al. [33] summarized the usage of EPO based on previous literatures. Most of the studies prefer to give 30,000~40,000 IU/w subcutaneously and the regime start 3–4 weeks before surgery. Considering the lack of patient compliance before hospitalization, we made some changes in the administration of rHu-EPO during the hospital stay to make it more convenient and easily controlled compared with previous studies. In our study, 10,000 IU rHu-EPO was injected subcutaneously on the operation day and postoperative 1–7 days. The total dosage of rHu-EPO requirements in our regimen is less than previous studies. However, our results were consistent with previous reports and the regime could also be assessed as an alternative to reducing EPO dose requirements.

Several limitations of this study should be noted. First, a major limitation is the lack of a sufficiently large patient sample in our study but we found the false negative rate of VTE examined by TEG is extremely low. Second, we could not evaluate the long-term influence of rHu-EPO on VTE as the treatment was too short, but the duration of anticoagulant therapy is much longer than the half-life of rHu-EPO subcutaneous injection. Finally, all participators received tranexamic acid and the oral anticoagulant rivaroxaban, which might have impacts on hematological parameters. However, the potential influence did not affect the conclusion because the same drugs were applicable to both groups.

## Conclusions

Postoperative use of rHu-EPO and iron supplementation could improve the anemia-related parameters and reduce the requirement for ABT after THA without increasing the risk of VTE in the short term.

## Data Availability

There are available.

## Abbreviations

THA: Total hip arthroplasty
ABT: Allogenic blood transfusions
EPO: Erythropoietin
rHu-EPO: Recombinant human erythropoietin
VTE: Venous thromboembolism
DVT: Deep vein thrombosis
TEG: Thrombelastography
Pre-op: Pre-operation
POD: Postoperative day
RBC: Red blood cell
Hb: Hemoglobin
HCT: Hematocrit
PLT: Platelet
FDP: Fibrin degradation products
PT: Prothrombin time
APTT: Activated partial thromboplastin time
FIB: Fibrinogen
TT: Thrombin time
INR: International normalized ratio
R: Reaction time
MA: Maximum amplitude
CI: Coagulation index
FDA: Food and Drug Administration
